# IRW (Isoleucine–Arginine–Tryptophan) Improves Glucose Tolerance in High Fat Diet Fed C57BL/6 Mice via Activation of Insulin Signaling and AMPK Pathways in Skeletal Muscle

**DOI:** 10.3390/biomedicines10061235

**Published:** 2022-05-26

**Authors:** Stepheny C. de Campos Zani, Myoungjin Son, Khushwant S. Bhullar, Catherine B. Chan, Jianping Wu

**Affiliations:** 1 Department of Physiology, University of Alberta, Edmonton, AB T6G 2H7, Canada; zani@ualberta.ca (S.C.d.C.Z.); cbchan@ualberta.ca (C.B.C.); 2 Department of Agricultural Food & Nutritional Science, University of Alberta, Edmonton, AB T6G 2P5, Canada; mjson0823@gmail.com (M.S.); bhullar@ualberta.ca (K.S.B.); 3 Department of Pharmacology, University of Alberta, Edmonton, AB T6G 2H7, Canada

**Keywords:** ACE, bioactive peptides, IRW, insulin resistance, obesity

## Abstract

IRW (Isoleucine–Arginine–Tryptophan), has antihypertensive and anti-inflammatory properties in cells and animal models and prevents angiotensin-II- and tumor necrosis factor (TNF)-α-induced insulin resistance (IR) in vitro. We investigated the effects of IRW on body composition, glucose homeostasis and insulin sensitivity in a high-fat diet (HFD) induced insulin resistant (IR) model. C57BL/6 mice were fed HFD for 6 weeks, after which IRW was incorporated into the diet (45 or 15 mg/kg body weight (BW)) until week 14. IRW45 (at a dose of 45 mg/kg BW) reduced BW (*p* = 0.0327), fat mass gain (*p* = 0.0085), and preserved lean mass of HFD mice (*p* = 0.0065), concomitant with enhanced glucose tolerance and reduced fasting glucose (*p* < 0.001). In skeletal muscle, IRW45 increased insulin-stimulated protein kinase B (AKT) phosphorylation (*p* = 0.0132) and glucose transporter 4 (GLUT4) translocation (*p* < 0.001). Angiotensin 2 receptor (AT2R) (*p* = 0.0024), phosphorylated 5′-AMP-activated protein kinase (AMPKα) (*p* < 0.0124) and peroxisome proliferator-activated receptor gamma (PPARγ) (*p* < 0.001) were enhanced in skeletal muscle of IRW45-treated mice, as was the expression of genes involved in myogenesis. Plasma angiotensin converting enzyme-2 (ACE2) activity was increased (*p* = 0.0016). Uncoupling protein-1 in white adipose tissue (WAT) was partially restored after IRW supplementation. IRW improves glucose tolerance and body composition in HFD-fed mice and promotes glucose uptake in skeletal muscle via multiple signaling pathways, independent of angiotensin converting enzyme (ACE) inhibition.

## 1. Introduction

The pathophysiology of Metabolic syndrome (MetS) is complex involving obesity, IR, dyslipidemia, and hypertension, but oxidative stress and inflammation also contribute to its progression [1]. MetS also involves overactivation of the renin angiotensin system (RAS), which is linked to obesity and IR [2]. Besides the systemic RAS, an independent local RAS in skeletal muscle [3] influences insulin responsiveness [4]. ACE inhibitors or angiotensin II type 1 receptor (AT1R) blockers reduce IR in animal models and also improve insulin sensitivity in humans [5,6,7], supporting the link between RAS and IR. Also, RAS blockade reverses the deleterious effect of exogenous angiotensin II on skeletal muscle mitochondria and improves glycemic control in mice [8].

Food-derived bioactive peptides exert effects beyond their nutritional value and modulate physiological parameters in different tissues [9]. Previous research demonstrated the beneficial effects of egg-derived peptides and hydrolysates on glucose tolerance [10], adipogenic capacity [11], and osteoblast differentiation [12]. Some egg-derived bioactive peptides are ACE inhibitors [13] and ameliorate IR and glucose intolerance [14]. Of particular interest is the ovotransferrin-derived, ACE inhibitory peptide [15] tripeptide IRW (Isoleucine–Arginine–Tryptophan), which exhibits anti-inflammatory and antioxidant effects in endothelial cells [16,17], reduces blood pressure in rodents [18,19], and improves angiotensin II- or TNF-α-induced IR in a skeletal muscle cell line [20,21]. However, whether IRW has glucoregulatory properties in vivo is unknown.

In this study, we investigated the insulin sensitizing effects of IRW in vivo using a high fat diet (HFD)-induced obese-IR mouse model. Because of the intimate crosstalk between obesity, hypertension, and IR, we hypothesized that IRW supplementation improves glucose intolerance by inhibiting RAS locally in skeletal muscle to improve insulin signaling.

## 2. Materials and Methods

### 2.1. Animals, Diet, and Body Weight (BW) Measurements

The animal experimental protocol was approved by the Animal Care and Use Committee of the University of Alberta (Protocol# 1402) in accordance with guidelines issued by the Canadian Council on Animal Care and followed the ARRIVE guidelines. Thirty-two male 4-week-old C57BL/6 mice were purchased from Charles River Canada (St. Constant, QC, Canada) and housed 2 per cage with *ad libitum* access to standard food and water, a 12:12-h cycle of light:dark with 60% humidity and 23 °C temperature. Eight mice were fed with low fat diet (LFD, 10% kcal from fat, Envigo, Indianapolis, IN, USA, TD06415) and the remainder with HFD (45% kcal from fat, Envigo TD110675) for 6 weeks. Diet composition is shown in Table 1. The initial 6 weeks of HFD were used to induce obesity, confirmed by at least a 20% increase in BW and significantly higher fat mass and lower lean mass compared to low fat diet (LFD) fed animals (Supplementary Table S1). Animals were then divided into 4 groups: LFD control, HFD control, high dose IRW + HFD (IRW45; at a dose of 45 mg/kg BW), low dose IRW + HFD (IRW15; at a dose of 15 mg/kg BW) (n = 8/group). These diets continued for another 8 weeks with *ad libitum* access to food and water. In total, mice consumed HFD for 14 weeks to induce obesity and glucose intolerance [22]. Food consumption was measured once every 3 days and BW twice weekly. Body composition was evaluated using magnetic resonance imaging (MRI), Echo MRI^TM^ (Echo Medical Systems LLC, Houston, TX, USA) at week 6 and week 14.

### 2.2. IRW Dosage

The estimated doses of IRW used were 45 mg/kg BW and 15 mg/kg BW. The dosages were selected based on previous studies done by our group in spontaneous hypertensive rats [18,19] and cell line studies [15,16]. The peptide was administered mixed in the animal’s diet starting at week 7 of the 14-week trial, and the animals had *ad libitum* access to food and water. IRW was synthesized by Genscript (Piscataway, NJ, USA) with ≥98% purity.

### 2.3. Oral Glucose Tolerance and Insulin Tolerance Tests

At weeks 12 and 13, respectively, ITT and OGTT were performed [23] with the following modifications: For ITT, 1.5 IU/kg BW insulin was injected intraperitoneally. For OGTT, 70% glucose solution was used, and 1 g of glucose/kg BW was given via oral gavage. In both cases, blood glucose was measured for up to 2 h. Blood glucose was measured from the tail vein using a Contour^®^Next glucometer (Mississauga, ON, Canada). Fasting glucose and fasting insulin were used to calculate homeostatic model assessment insulin resistance (HOMA-IR) using the formula: (fasting glucose (mmol/L)) × (fasting insulin (μU/mL))/22.5). OGTT was the primary outcome assessed in this study.

### 2.4. Tissue Collection

At week 14, all animals were fasted for 16 h and injected with insulin (2 IU/kg BW) intraperitoneally to stimulate insulin signaling 10 min prior to euthanasia. Animals were euthanized using CO_2_ and blood was collected via cardiac puncture. Plasma, gastrocnemius skeletal muscle and WAT from retroperitoneal and epidydimal depots were collected, snap frozen, and stored at −80 °C until further analysis.

### 2.5. Protein Extraction and Western Blotting

Total protein from skeletal muscle was extracted using a lysis buffer containing phosphatase and protease inhibitors. WAT total protein extraction was performed using a commercial kit (Invent Biotechnologies Inc., Plymouth, MA, USA). Plasma membrane protein was extracted using a commercial kit (Thermo Fisher Scientific, Waltham, MA, USA) [20]. Total protein content was measured using the bicinchoninic acid assay. Western blotting was performed as previously [10] with the following modifications: a 9% sodium dodecyl sulfate polyacrylamide gel electrophoresis was run and protein was transferred to a nitrocellulose membrane, which was incubated overnight with antibodies against p-AKT (Cell Signaling Technology, Beverly, MA, USA), AKT (Santa Cruz Biotechnologies Inc., Dallas, TX, USA), GLUT4 (Abcam, Toronto, ON, Canada), ACE, ACE2, AT1R, AT2R, Mas receptor, PPAR-γ, mammalian target of rapamycin (mTOR), p-mTOR, AMPKα, p-AMPKα (Thr172), P70 S6 kinase (S6K), p-P70 S6K (Thr389), uncoupling protein (UCP)-1 (Sigma, St. Louis, MO, USA), β-actin (Sigma) and glyceraldehyde-3-phosphate dehydrogenase (GAPDH). After incubating with appropriate fluorescent-conjugated secondary antibodies (Li-cor Biosciences, Lincoln, NE, USA) for 1 h at room temperature, protein bands were quantified by densitometry using Image Studio Lite 5.2 (Li-Cor Biosciences).

### 2.6. Plasma RAS Components and Insulin

Plasma concentrations of RAS components were quantified by mouse specific commercial ELISAs: ACE (Aviva System, San Diego, CA, USA), ACE2 (Abcam), angiotensin II (Enzo Life Sciences, Burlington, ON, Canada), angiotensin (1–7) (Aviva System). Insulin was measured using an ELISA from (Abcam, Toronto, ON, Canada).

### 2.7. RNA Sequencing and Quantitative RT-PCR (qPCR)

Total RNA was extracted from skeletal muscle using TRIzol reagent (Invitrogen, Life Technologies Inc., Burlington, ON, Canada) and quantified by measuring the absorbance at 260 nm and purity assessed by the A260/280 ratio. Total RNA (500 ng, with RNA integrity number > 8 for all samples) was used for the preparation of RNAseq libraries with the NEBNext Ultra II Directional RNA Library Prep Kit from Illumina (NEB, Mississauga, ON, Canada). Enriched mRNA was reverse-transcribed and second-strand cDNA synthesis was performed. Double-stranded cDNAs were A-tailed to enable adapter ligation and, finally, libraries were indexed by 15 PCR cycles. Libraries were sequenced on a NextSeq 500 instrument (Illumina), following a paired-end 150 cycle protocol. Deregulated transcripts were annotated using the BioMart database from Ensembl (EMBL-EBI Hinxton, Cambridgeshire, UK).

For qPCR, cDNA was synthesized from 1 μg total RNA using the High-Capacity cDNA Reverse Transcription Kit (Thermo Fisher Scientific, Waltham, MA, USA). mRNA expression of target genes was determined by real-time qPCR using GAPDH as the endogenous control. All of the qPCR experiments and analyses were conducted using the MIQE guidelines [24]. The primers were designed based on the genomic sequence deposited in GenBank are described in Table S2.

### 2.8. Statistics and Sample Size

All data presented were expressed as mean ± SEM of 5–8 mice from each treatment group as indicated in the figure and table legends. Statistical analysis was performed using GraphPad Prism 7.0 (San Diego, CA, USA). Outliers indicated by the statistical software were removed prior to data evaluation. Data were evaluated by one-way ANOVA, Kruskal–Wallis’ test, or two-way ANOVA when appropriate. HFD group was set as the control group for all the analysis because our intention was to evaluate the impact of a HFD supplemented with peptide compared to HFD alone. For RNA sequencing, transcripts were considered differentially expressed when they had a corrected *p*-value < 0.05. Post hoc analysis was done using Bonferroni’s or Dunn’s test. *p*-value was considered significant if <0.05.

## 3. Results

### 3.1. Food Intake and Body Composition

No sustained differences in food intake were detected between the groups (Supplementary Figure S1). At week 14 the LFD group had lower BW than the HFD group (*p* < 0.001). Of the peptide treatments, only the high dose of IRW (IRW45) reduced final BW (*p* = 0.0327) and both absolute (g) and relative (%) BW gain (*p* < 0.001). Moreover, the IRW45 group presented lower absolute (g) (*p* < 0.001) and relative (%) fat mass gain (*p* = 0.0085), and less relative (%) lean mass loss (*p* = 0.0065) compared to the HFD group (Table 2). BW and composition changes were not seen with IRW15.

### 3.2. Glucose Homeostasis and Plasma Insulin

**Figure 1:**
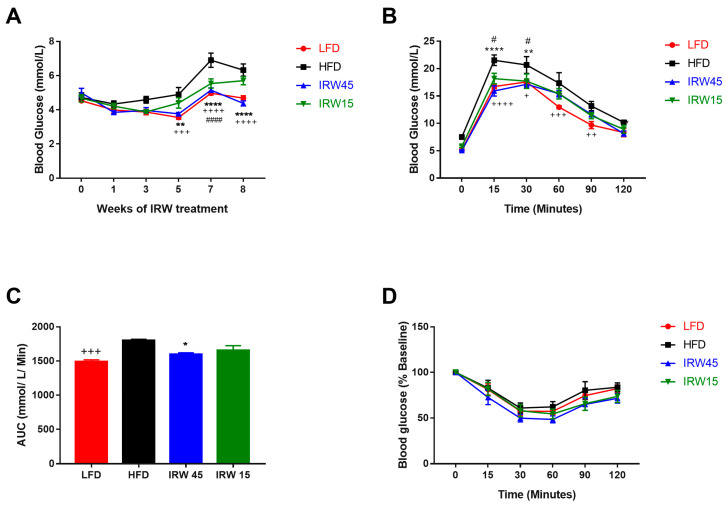
Glucose homeostasis after IRW supplementation. (**A**) Fasting glucose over time (n = 6–8), (**B**) Oral glucose tolerance test (OGTT) (n = 6–8). (**C**) Area under the curve (AUC) for OGTT (n = 5–8). (**D**) Insulin tolerance test (ITT) as percentage of the baseline glucose values (n = 8). Data expressed as mean ± SEM and analyzed by two-way ANOVA (**A**,**C**,**D**) or one-way ANOVA (**B**) followed by Bonferroni’s post-hoc comparison test. * *p* < 0.05, ** *p* < 0.01 and **** *p* < 0.0001 between IRW45 and HFD. # *p* < 0.05 and #### *p* < 0.0001 between IRW15 and HFD. + *p* < 0.05, ++ *p* < 0.01, +++ *p* < 0.001 and ++++ *p* < 0.0001 between LFD and HFD.

The IRW45 group had lower fasting blood glucose beginning at week 9 of treatment compared to the HFD group (Figure 1A) and two-way ANOVA analysis showed significant interaction (*p* < 0.001) and diet effects (*p* < 0.001). At week 14, the LFD and IRW45 groups had lower fasting plasma insulin concentration (*p* < 0.001) and fasting blood glucose (*p* < 0.001) compared to the HFD (Table 2). HOMA-IR was lower in LFD (*p* < 0.0001) and IRW45 (*p* < 0.001) groups compared to HFD. However, HOMA-IR from the IRW15 did not differ from the HFD group (Table 2). Two-way ANOVA showed a diet effect on OGTT (*p* = 0.001). Both doses of IRW lowered circulating glucose at 15 and 30 min compared with HFD (Figure 1B). The area under the curve (AUC) was lower in the LFD (*p* < 0.001) and IRW45 (*p* = 0.0195) compared to the HFD group (Figure 1C). Despite changes in ITT when expressed as absolute glucose concentrations (Supplementary Figure S2), no significant differences between the groups were observed when data were adjusted to baseline blood glucose concentration (Figure 1D).

### 3.3. Insulin Signaling and PPARγ Abundance

**Figure 2:**
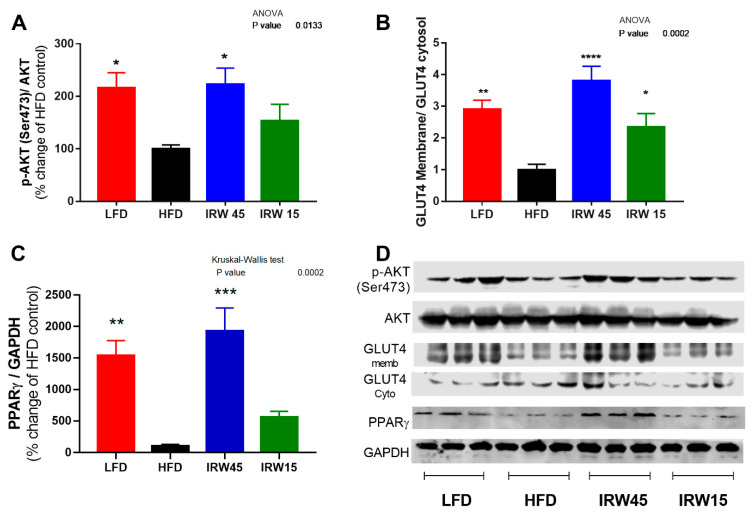
Skeletal muscle insulin signaling and PPARγ abundance. (**A**) p-AKT, (**B**) GLUT4 membrane/cytosol, (**C**) PPARγ, and (**D**) representative blots. p-AKT was normalized to total AKT. GLUT4 is expressed as a ratio of membrane to cytosolic GLUT4. PPARγ was normalized to GAPDH. Data expressed as mean ± SEM of n = 6. Analysis by one-way ANOVA followed by Bonferroni’s post-hoc test or Kruskal–Wallis followed by Dunn’s post-hoc test. * *p* < 0.05, ** *p* < 0.01 *** *p* < 0.001 and **** *p* < 0.0001 versus HFD. AKT, Protein kinase B; PPARγ, Peroxisome proliferator-activated receptor gamma; GLUT4, glucose transporter 4.

Insulin-stimulated phosphorylation of AKT (Ser473) in muscle was higher in LFD (*p* = 0.0196) and IRW45 (*p* = 0.0132) compared to the HFD group, whereas IRW15 was non-significantly increased (Figure 2A). Consistent with this result, GLUT 4 translocation to the plasma membrane in skeletal muscle was ~4-fold higher in the IRW45 (*p* < 0.001) and 2-fold higher in IRW15 (*p* = 0.04) compared to the HFD group, as shown by the ratio of membrane GLUT4/cytosol GLUT4 (Figure 2B). In addition, PPARγ abundance was 15–17-fold higher in LFD (*p* = 0.0013) and IRW45 (*p* < 0.001) compared to HFD (Figure 2C).

In both retroperitoneal and epididymal WAT, no changes in AKT phosphorylation or PPARγ abundance were seen following IRW supplementation (Supplementary Figure S3A–F).

### 3.4. RAS Components

**Figure 3:**
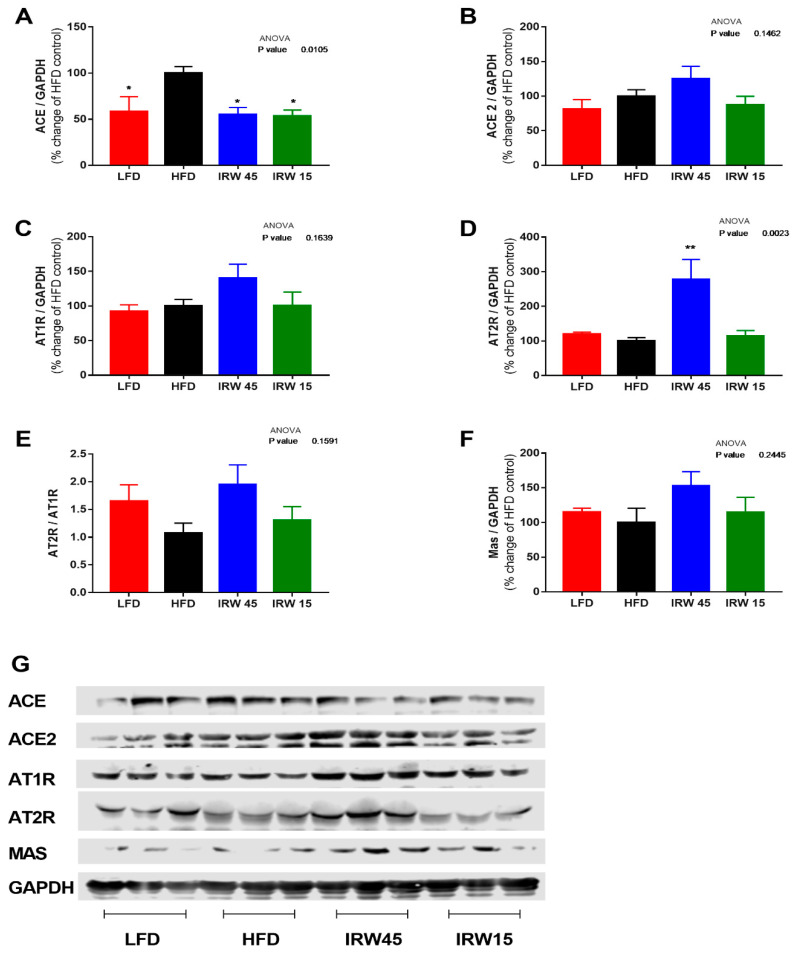
Skeletal muscle renin angiotensin system components. (**A**) ACE, (**B**) ACE2, (**C**) AT1R, (**D**) AT2R, (**E**) AT2R/AT1R ratio, (**F**) Mas receptor, and (**G**) representative blot. ACE, ACE2, AT1R, AT2R, and Mas were normalized to GAPDH. Data expressed as mean ± SEM of n = 5–6 mice. Analysis by one-way ANOVA followed by Bonferroni’s post-hoc test. * *p* < 0.05 and ** *p* < 0.01 versus HFD. ACE, angiotensin converting enzyme; ACE2, angiotensin converting enzyme 2; AT1R, angiotensin receptor type 1; AT2R, angiotensin receptor type 2.

**Figure 4:**
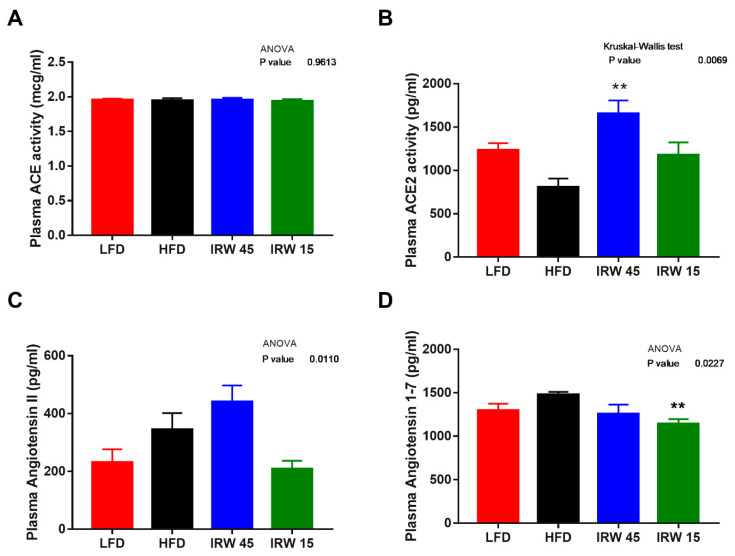
Plasma renin angiotensin system components. (**A**) Plasma angiotensin converting enzyme (ACE), (**B**) Plasma angiotensin converting enzyme 2 (ACE2), (**C**) Plasma angiotensin II, and (**D**) Plasma angiotensin (1–7). Data expressed as mean ± SEM of n = 4–7 mice. Analysis by one-way ANOVA followed by Bonferroni’s post-hoc comparison test or Kruskal–Wallis followed by Dunn’s post hoc test when appropriate. ** *p* < 0.01 versus HFD.

In skeletal muscle, there were no significant differences in ACE2, AT1R, and Mas receptor between the groups (Figure 3B,C,F). However, a lower ACE abundance in LFD (*p* = 0.0259), IRW15 (*p* = 0.0157), and IRW45 (*p* = 0.0113) groups was observed compared to HFD (Figure 3A). In addition, AT2R abundance increased almost 3-fold in the IRW45 group compared to HFD (*p* = 0.0024) (Figure 3D). The plasma ACE activity was similar between groups (Figure 4A). Although ANOVA analysis identified an overall effect (*p* = 0.01) on plasma angiotensin II, post-hoc analysis showed no significant differences between groups (Figure 4C). Whereas ACE2 activity was highest in the IRW45 group (*p* = 0.0016) (Figure 4B), while plasma Ang (1–7) plasma concentration was not increased by IRW compared to the HFD (Figure 4D).

### 3.5. Skeletal Muscle Gene Expression

**Figure 5:**
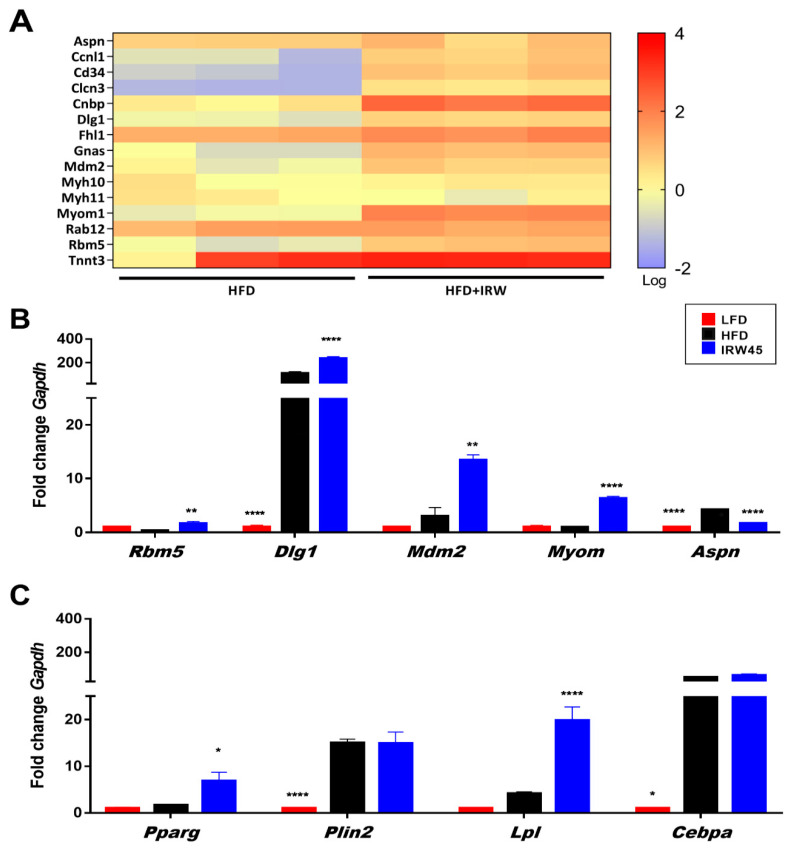
Skeletal muscle gene expression of mice fed IRW for 8 weeks. (**A**) Heatmap showing the abundance of major genes involved in muscle synthesis modulated by IRW45 determined by RNA sequencing. (**B**) *Rbm5*, *Mdm2*, *Dlg1*, *Myom1*, and *Aspn* qPCR validation of IRW in vivo using gastrocnemius skeletal muscle. Data expressed as mean ± SEM of n = 5 mice (**C**) *Pparg, Plin2, Cebpa* and *Lpl* qPCR validation using gastrocnemius skeletal muscle. Data expressed as mean ± SEM of n = 5 mice. Analysis by one-way ANOVA followed by Bonferroni’s post-hoc comparison test or Kruskal–Wallis followed by Dunn’s post hoc test when appropriate. * *p* < 0.05, ** *p* < 0.01, and **** *p* < 0.0001 versus HFD.

RNA sequencing of gastrocnemius muscle transcripts revealed that IRW45 elicited a generally higher abundance of transcripts related to muscle synthesis (Figure 5A). qPCR validation identified *Rbm5* (*p* = 0.0094), *Mdm2* (*p* = 0.0094), *Dlg1* (*p* < 0.001), and *Myom1* (*p* < 0.001) genes upregulated and *Aspn* (*p* < 0.001) downregulated by IRW45 compared to the HFD group (Figure 5B). For PPARγ related genes, IRW45 significantly enhanced the expression of *Pparg* (*p* = 0.0112) and *Lpl* (*p* < 0.001) but not *Plin2* or *Cebpa* (Figure 5C). However, it should be noted that the expression of *Plin2* and *Cebpa* were 15 and ~50-fold higher in HFD and IRW45 than LFD.

### 3.6. AMPKα Abundance and mTOR Signaling

**Figure 6:**
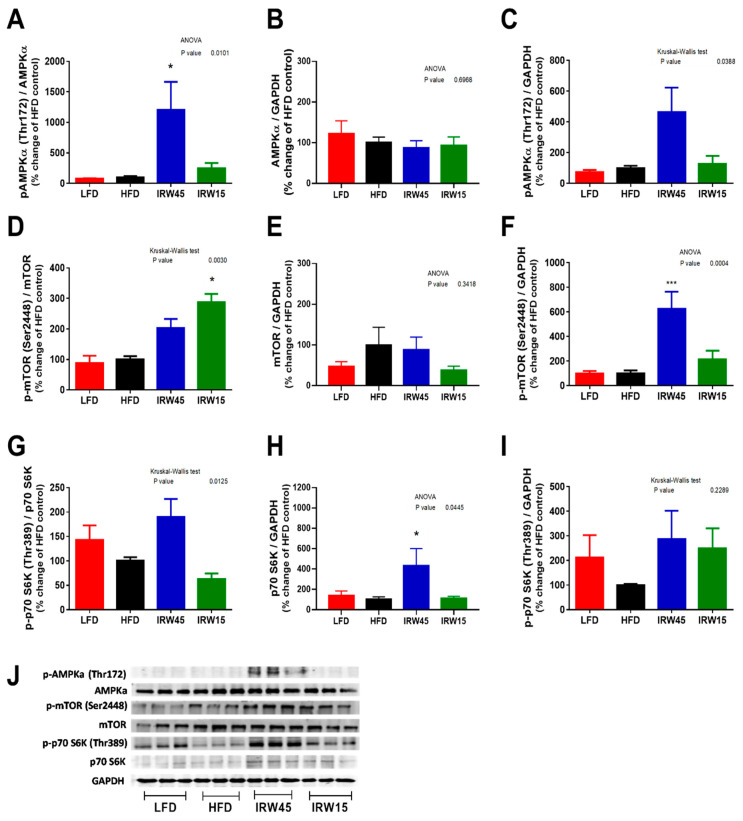
Skeletal muscle AMPKα, mTOR and P70 S6K protein abundance. (**A**) p-AMPKα/AMPKα. (**B**) AMPKα/GAPDH. (**C**) p-AMPKα/GAPDH. (**D**) p-mTOR/mTOR. (**E**) mTOR/GAPDH. (**F**) p-mTOR/GAPDH. (**G**) p-P70 S6K/P70 S6K. (**H**) P70 S6K/GAPDH. (**I**) p-P70 S6K/GAPDH and (**J**) representative blots. Data expressed as mean ± SEM of n = 5–6 mice. Analysis by one-way ANOVA followed by Bonferroni’s post-hoc comparison test or Kruskal–Wallis followed by Dunn’s post hoc test when appropriate. * *p* < 0.05 and *** *p* < 0.001 versus HFD. AMPK, 5′ AMP-activated protein kinase; mTOR, mammalian target of rapamycin; P70 S6K, Ribosomal protein S6 kinase beta-1.

IRW45 treatment elicited a ~10-fold increase in AMPKα phosphorylation (Thr172) relative to total AMPKα in skeletal muscle compared to HFD (*p* < 0.0124) (Figure 6A), with no change in total AMPKα (Figure 6B). ANOVA indicated an overall diet effect in p-AMPKα over GAPDH (Figure 6C), consistent with findings in Figure 6A. No changes in total or phosphorylated AMPKα abundance in retroperitoneal or epididymal WAT were observed after IRW supplementation (Supplementary Figure S4 and Figure S5, respectively).

Phosphorylated (Ser2448) mTOR relative to total mTOR had an overall significant increase (*p* = 0.003) in skeletal muscle, but the post-hoc analysis revealed no statistical significance between IRW45 and HFD (*p* = 0.2332), while in IRW15 p-mTOR (Ser2448) was increased (*p* < 0.0138) (Figure 6D). Total mTOR was not affected (Figure 6E); however, p-mTOR was statistically increased by IRW45 (*p* < 0.001) (Figure 6F). The downstream p70 S6K protein phosphorylation trended to elevated IRW45 relative to total p70 S6K (Figure 6G, *p* = 0.2075) and 3-fold relative to GAPDH (Figure 6I, *p* = 0.15); however, total p70 S6K protein was significantly increased (*p* < 0.045) in IRW45 compared to HFD (Figure 6H). In adipose tissue, no changes were seen in total and phosphorylated mTOR or p70 S6K in both retroperitoneal and epididymal WAT (Supplementary Figure S4 and Figure S5, respectively).

### 3.7. Adipose Tissue UCP-1 Abundance

**Figure 7:**
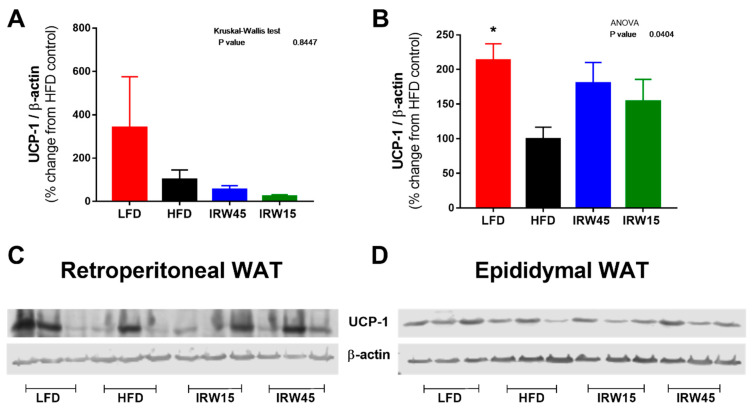
White adipose tissue UCP-1 protein abundance. Retroperitoneal WAT UCP-1 (**A**) and representative blot (**C**). Epidydimal WAT UCP-1 (**B**) and representative blot (**D**). UCP-1 was normalized to β-actin. Data expressed as mean ± SEM of n = 5–6 mice. Analysis by one-way ANOVA followed by Bonferroni’s post-hoc test or Kruskal–Wallis followed by Dunn’s post hoc test when appropriate. * *p* < 0.05 versus HFD. UCP, uncoupling protein.

The investigation of UCP-1 abundance in retroperitoneal and epididymal WAT is shown in Figure 7. UCP-1 was decreased by HFD compared with LFD and this was significant in epididymal WAT (*p* < 0.0188) (Figure 7B). IRW15 and IRW45 exhibited intermediate abundance in epididymal WAT.

## 4. Discussion

Natural health products [25] are used by a wide range of the population but their efficacy in managing complex metabolic diseases is still debated. Despite that, food-derived bioactive peptides exhibit positive physiological effects related to metabolic diseases and their complications [14,26,27]. IRW is an ovotransferrin-derived bioactive peptide previously shown to exert antihypertensive and anti-inflammatory effects [17,19]. In addition, in vitro, IRW presented antioxidant effects and improved insulin signaling [20,21]. In this study, using an obese, IR rodent model we demonstrated that IRW supplementation at a dose of 45 mg/kg BW: (1) prevented BW and fat mass gain during HFD treatment while protecting lean body mass; (2) improved glucose tolerance and fasting blood glucose and insulin concentrations; and (3) enhanced insulin-dependent and -independent signaling governing glucose uptake in skeletal muscle. IRW15 was not as effective as IRW45, illustrating dose dependence. For this reason, the discussion is focused on IRW45 findings. Contrary to our hypothesis, these activities of IRW did not appear to involve the inhibition of local RAS.

As previously demonstrated [18,19], IRW retained biological activity in vivo possibly because we mixed IRW into the HFD, which may have protected from degradation by digestive enzymes [28]. However, IRW can be degraded into the dipeptide IR in simulated gastrointestinal digestion, which decreases its ACE inhibitory activity drastically in vitro [15]. We did not calculate the concentration or characterize the bioactive form reaching the bloodstream in the current study and we cannot exclude the possibility of the dipeptide IR being bioactive in vivo, therefore it is only a speculation. Nevertheless, IRW at a dosage of 45 mg/kg BW promoted enhanced glucose homeostasis. This dosage is comparable to other studies investigating the role of small-molecules, natural products or bioactive peptides in cardio-metabolic conditions using rodent models. For example, AdipoRon, a small-molecule agonist of adiponectin receptor is used at 50 mg/kg BW [29], curcumin at 100 mg/kg BW [30], soy β-Conglycin at 10% of diet (*w*/*w*) [31] and the di-peptide (Trp-His) at dosages ranging between 10–100 mg/kg BW [32,33]. Moreover, metformin is used at 200 mg/kg BW in rodent models of diabetes [34,35].

In this study, IRW45 improved both fasting and insulin-stimulated glucose indices and decreased fasting insulin in HFD-fed, glucose-intolerant mice, consistent with reduced HOMA-IR. Similarly, an egg white hydrolysate improved glucose tolerance and insulin sensitivity in HFD rats [10]. In Zucker Fatty rats, egg white hydrolysate treatment lowered fasting insulin but not glucose concentrations, resulting in reduced HOMA-IR and HOMA-β indices [36]. Although our ITT study shows that IRW45 treatment improved insulin sensitivity compared to the HFD group, the significance was lost after adjustment for baseline blood glucose concentration, suggesting that the effects observed were dependent on differences in fasting blood glucose concentration.

Glucose uptake in skeletal muscle occurs via insulin-dependent and -independent pathways. Insulin activates the PI3K-AKT cascade leading to translocation of GLUT4 to the plasma membrane, and thus increases glucose uptake [37], which was demonstrated in skeletal muscle of IRW45 treated animals compared to the HFD. However, because ITT was not different between groups, insulin-independent pathways may also play a role. AMPK activation, such as in muscle contraction, enhances GLUT4 translocation [38] and increases glucose entry independently of insulin [39]. Indeed, AMPKα phosphorylation in skeletal muscle of IRW45 animals was significantly increased. Both AKT and AMPK pathways could contribute to improved glucose tolerance observed in IRW45 mice. We also acknowledge the possibility of enhanced basal AKT phosphorylation by IRW directly. In this study, only insulin-stimulated animals were included, which did not allow for this latter analysis.

We initially hypothesized that IRW-mediated improvements in insulin signaling would be associated with reduced local RAS activity, based on previous studies [15,18,19,40]. Despite IRW being an ACE inhibitor in vitro [15], we found no effect of IRW on systemic ACE activity in this IR model, similar to previous results using IRW in SHR rats [18]. However, IRW reduced ACE protein abundance in skeletal muscle, which might contribute to lower local ACE activity. Moreover, plasma ACE2 activity was increased in our study, consistent with previous studies showing that oral IRW supplementation enhanced circulating ACE2 abundance and activity [18], and ACE2 protein expression in the aorta of SHR rats [40]. ACE2 antagonizes the actions of angiotensin II, thereby reducing blood pressure, and reducing CVD risk through angiotensin (1–7)/Mas receptor axis as reviewed [41]. Interestingly, in this study angiotensin (1–7) was not increased by IRW treatment.

In skeletal muscle, modulation of AT1R and AT2R regulates insulin action locally, with systemic AT2R blockade impairing insulin-stimulated AKT phosphorylation, whole body glucose uptake, and muscular microvascular function, while systemic AT1R blockade restored muscle insulin signaling [4]. AT2R opposes the effects of AT1R activation in blood vessels with their interplay regulating blood flow and glucose utilization in skeletal muscle [42]. In our study, IRW45 increased AT2R abundance in skeletal muscle. Similarly, we previously showed that egg hydrolysate enhanced AT2R abundance in WAT and liver, and improved glucose tolerance [10]. Possibly, increased AT2R abundance in skeletal muscle tissue permits increased binding of angiotensin II to AT2R in the capillary endothelium, thus improving blood flow to facilitate insulin access to muscle cells and enhancing glucose uptake. Moreover, IRW may directly activate AT2R in muscle cells, which we speculate may improve glucose transport via AMPK and PPARγ activation. Despite no clear direct link between AT2R and AMPK yet being demonstrated, RAS modulation improves glucose tolerance and insulin sensitivity via AMPK activation [43,44].

In some tissues, such as WAT, AT2R is linked to PPARγ as evidenced by PPARγ mRNA and activation being enhanced by AT2R agonists [45] and egg white hydrolysate concomitantly increased AT2R and PPARγ abundance [10]. Thiazolidinediones (TZDs) are PPARγ agonists and cause insulin sensitizing effects by enhancing skeletal muscle glucose uptake, reducing liver glucose output, and affecting WAT physiology [46]. PPARγ agonists potentiate AKT phosphorylation in WAT and skeletal muscle [47] and specific deletion of PPARγ in skeletal muscle of mice induces IR [48]. In IR hamsters, PPARγ RNA expression in skeletal muscle is downregulated, along with other genes regulated by PPARγ such as *Ppargc1a*, *Lpl*, and *Adipoq* (adiponectin) genes [49]. In this study, IRW45 treatment upregulated *Pparg* and *Lpl* in skeletal muscle while increasing PPARγ protein abundance, suggesting that IRW may upregulate a cassette of PPARγ-related genes as part of its metabolic activity.

Despite similar caloric intake, IRW45 improved body composition by reducing BW and fat mass gain, while protecting lean mass. In humans, the reduction of whole-body fat mass after an exercise intervention was associated with increased insulin sensitivity index [50]. Recently, an extract from rice hulls decreased fat mass by suppressing adipogenic genes in epididymal WAT and liver while enhancing AMPKα protein, consistent with increased fatty acid oxidation [51]. However, neither AMPKα nor PPARγ abundance changed in visceral WAT after IRW treatment. We also investigated whether enhanced thermogenesis in WAT induced by IRW might explain reduced fat mass because AT2R activation was previously shown to induce UCP-1 in epididymal WAT [52] and brown adipose tissue [53]. We found that compared with LFD, UCP-1 was reduced in HFD epididymal WAT, similar to other findings [54]. IRW45 treatment tended to increase UCP-1 but not as strongly as expected if thermogenesis was the main route eliciting fat mass loss. The mechanism by which IRW reduced fat mass is still unclear and other pathways deserve investigation.

Skeletal muscle synthesis is a key indicator of metabolic health and is regulated by insulin [55]. Upregulation of genes involved in muscle synthesis was induced by IRW. Primarily, mTOR activation is modulated by nutrients and, once activated, is involved in protein and lipid synthesis [56]. However, despite increased AMPK phosphorylation and expression of muscle synthesis genes in skeletal muscle, phosphorylation of mTOR (Ser2448) by IRW was not dose-dependent nor correlated with phosphorylation of the downstream S6K P70. Nevertheless, the gene upregulation observed after IRW supplementation indicates a possible ability of IRW to trigger myogenesis pathways, which may be related to our observation of protected lean body mass. Alternatively, IRW may be acting independently of mTOR to promote these effects.

In summary, IRW reduced BW and fat mass gain while improving glucose tolerance and insulin sensitivity in HFD mice. We identified several mechanisms of action for IRW in skeletal muscle and, to a lesser extent, WAT that were independent of ACE inhibition. Pathways influenced by IRW include the AKT/GLUT4 and AMPKα/GLUT4, which both enhance glucose uptake in skeletal muscle, while activation of the AT2R/PPARγ pathway could improve insulin sensitivity. Furthermore, IRW may reduce inflammation [18,21], contributing to insulin sensitization. Because the liver regulates fasting glucose homeostasis, IRW may also improve liver insulin sensitivity during fasting, leading to better glucose tolerance. IRW effects in the liver are currently being investigated by our group. Thus, IRW has the potential to exert beneficial effects on glucose homeostasis, making it a strong candidate to be further studied in the context of metabolic diseases.

## Figures and Tables

**Table 1:** Diet composition.

	LFD	HFD	IRW15	IRW45
Casein (g/kg)	210.0	245.0	245.0	245.0
L-Cystine (g/kg)	3.0	3.5	3.5	3.5
Corn Starch (g/kg)	445.0	85.0	85.0	85.0
Maltodextrin (g/kg)	50.0	115.0	115.0	115.0
Sucrose (g/kg)	160.0	200.0	200.0	200.0
Lard (g/kg)	20.0	195.0	195.0	195.0
Soybean Oil (g/kg)	20.0	30.0	30.0	30.0
Cellulose (g/kg)	37.15	58.0	58.0	58.0
Mineral Mix, AIN-93G-MX (94046) (g/kg)	35.0	43.0	43.0	43.0
Calcium Phosphate, dibasic (g/kg)	2.0	3.4	3.4	3.4
Vitamin Mix, AIN-93-VX (94047) (g/kg)	15.0	19.0	19.0	19.0
Choline Bitartrate (g/kg)	2.75	3.0	3.0	3.0
IRW (mg/kg BW)	n/a	n/a	15	45

**Table 2:** Body composition and metabolic profile of mice supplemented with IRW. Analysis by one-way ANOVA followed by Bonferroni’s post hoc test (lean mass change, fasting glucose initial and HOMA-IR) or Kruskal–Wallis’s test followed by Dunn’s post-hoc test. Data expressed as mean ± SEM of n = 7–8 mice. Values in the same row represented by different letters are statistically different (*p* ≤ 0.05) compared to HFD control.

	LFD	HFD	IRW45	**IRW15**
Body composition				
BW week 6 (g)	28.1 ± 0.8 ^a^	31.3 ± 0.7 ^a^	30.9 ± 0.9 ^a^	31.7 ± 1.0 ^a^
BW week 14 (g)	35.0 ± 0.7 ^a^	41.6 ± 0.8 ^b^	36.9 ± 0.5 ^a^	40.4 ± 1.0 ^b^
BW gain (g) (week 6–14)	7.0 ± 0.2 ^a^	10.2 ± 0.3 ^b^	6.0 ± 0.6 ^a^	8.6 ± 0.6 ^b^
BW gain (% of week 6)	25.1 ± 1.2 ^a^	32.8 ± 1.3 ^b^	19.9 ± 2.3 ^a^	27.5 ± 2.2 ^b^
Fat mass gain (g)	3.9 ± 0.5 ^a^	7.0 ± 0.3 ^b^	3.6 ± 0.4 ^a^	6.1 ± 0.4 ^b^
Fat mass gain (% BW)	8.2 ± 1.1 ^b^	9.5 ± 0.8 ^b^	4.8 ± 0.9 ^a^	8.9 ± 0.9 ^b^
Lean mass change (g)	0.8 ± 0.3 ^a^	2.6 ± 0.1 ^b^	2.0 ± 0.1 ^b^	2.3 ± 0.2 ^b^
Lean mass change (% BW)	−7.8 ± 1.0 ^b^	−9.2 ± 0.7 ^b^	−5.0 ± 0.9 ^a^	−7.9 ± 0.9 ^b^
Metabolic profile				
Fasting glucose week 6 (mmol/L)	4.5 ± 0.06 ^a^	4.7 ± 0.3 ^a^	5.0 ± 0.3 ^a^	4.7 ± 0.2 ^a^
Fasting glucose week 14 (mmol/L)	4.8 ± 0.1 ^a^	6.3 ± 0.4 ^b^	4.4 ± 0.2 ^a^	5.7 ± 0.2 ^b^
Fasting insulin week 14 (uU/mL)	192.4 ± 37.3 ^a^	780.0 ± 56.4 ^b^	383.1 ± 72.5 ^a^	623.7 ± 112.7 ^b^
HOMA-IR	5.7 ± 0.8 ^a^	32.0 ± 3.2 ^b^	11.0 ± 2.0 ^a^	23.0 ± 4.7 ^b^

## Data Availability

Data is contained within the article or supplementary material.

## Supplementary Materials

The following supporting information can be downloaded at: https://www.mdpi.com/article/10.3390/biomedicines10061235/s1, Figure S1: Food intake measured as kcal intake per day per animal during 8 weeks of IRW treatment. Figure S2: Insulin Tolerance test (ITT) and area under the curve (AUC) for OGTT after 8 weeks of IRW supplementation. Figure S3: White adipose tissue (WAT) insulin signaling and PPARγ protein abundance. Figure S4: Retroperitoneal white adipose tissue (WAT) AMPKα, mTOR and P70 S6K protein abundance. Figure S5: Epididymal white adipose tissue (WAT) AMPKα, mTOR and P70 S6K protein abundance. Table S1. Body composition of C57BL/6 mice after 6 weeks of LFD or HFD feeding. Table S2. Primers sequences used for RT-PCR assay.
