# Supplementary material for: IRW (Isoleucine–Arginine–Tryptophan) Improves Glucose Tolerance in High Fat Diet Fed C57BL/6 Mice via Activation of Insulin Signaling and AMPK Pathways in Skeletal Muscle
Source: Biomedicines. 2022 May 26;10(6):1235. doi: 10.3390/biomedicines10061235 (PMC9220315; doi:10.3390/biomedicines10061235)
Supplement: Supplementary file 1 [file biomedicines-10-01235-s001.zip › biomedicines-1744724-supplementary.pdf]

Supplementary Material

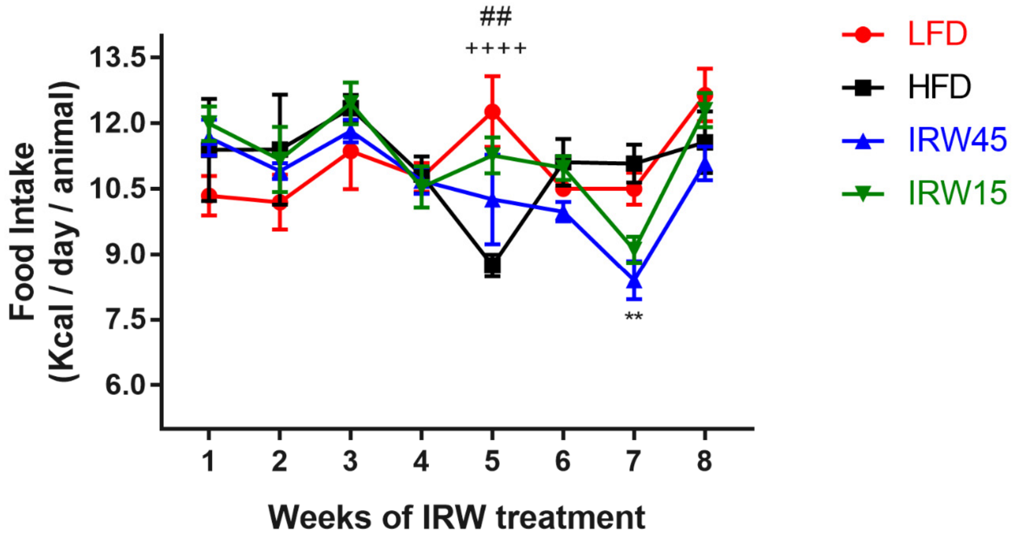

Figure S1: Food intake measured as kcal intake per day per animal during 8 weeks of IRW treatment. Data expressed as mean ± SEM of n=8 mice. Analysis by two-way ANOVA followed by Bonferroni's post-hoc comparison test. ** $p<0.01$ between IRW45 and HFD. ## $p<0.01$ between IRW15 and HFD. ++++ $p<0.0001$ between LFD and HFD.

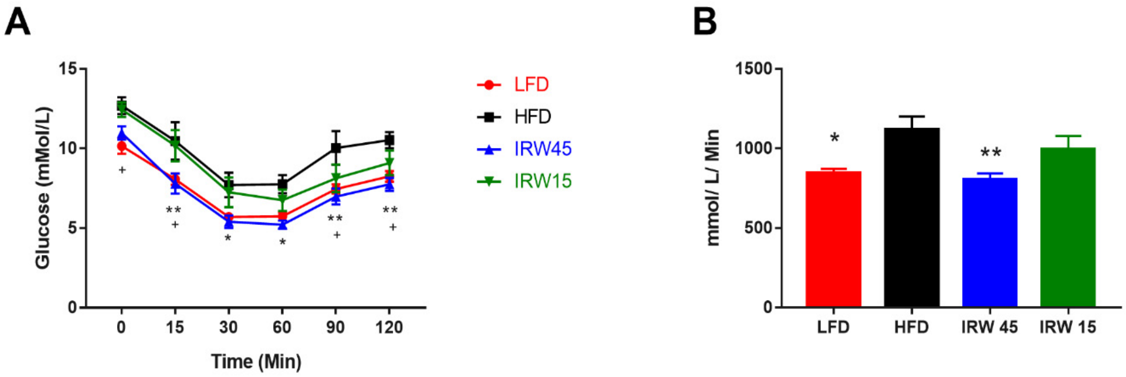

Figure S2: (A) Insulin Tolerance test (ITT) and **(B)** Area under the curve (AUC) for OGTT after 8 weeks of IRW supplementation (n= 8). Data expressed as mean $\pm$ SEM and analyzed by two-way ANOVA (A) or one-way ANOVA (B) followed by Bonferroni's post-hoc comparison test.* $p<0.05$ and ** $p<0.01$ between IRW45 and HFD. + $p<0.05$ between LFD and HFD.

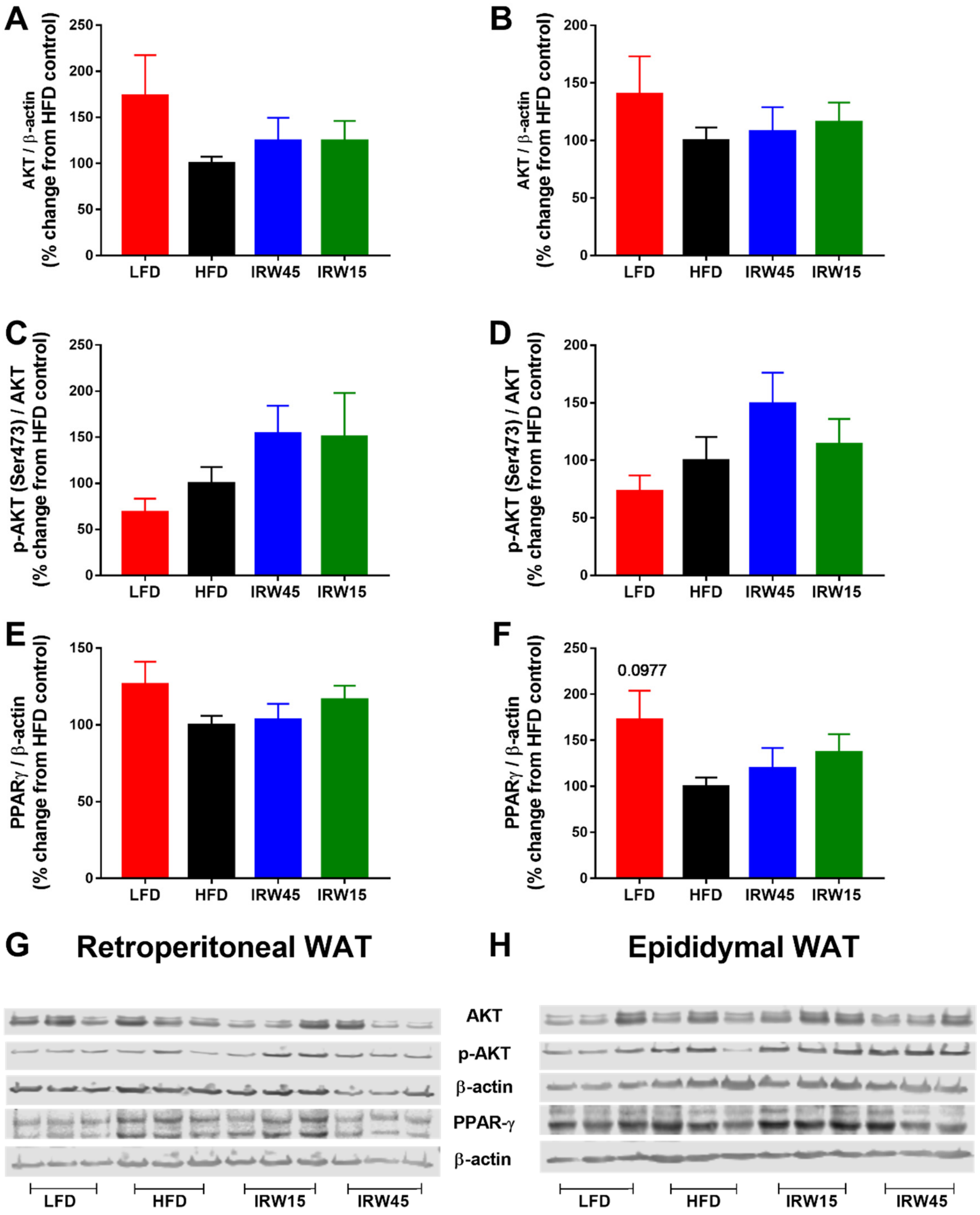

Figure S3: White adipose tissue (WAT) insulin signaling and PPARγ protein abundance. Retroperitoneal WAT **(A)**AKT, **(C)** p-AKT **(E)** PPARγ and **(G)** representative blots. Epidydimal WAT **(B)**AKT, **(D)** p-AKT, **(F)** PPARγ and **(H)** representative blots. p-AKT protein band was normalized to total AKT. PPARγ protein band was normalized to β-actin as the loading control. Data expressed as mean ± SEM of n= 6 mice. Analysis by one-way ANOVA followed by Bonferroni's post-hoc test; or Kruskal-Wallis test followed by Dunn's post hoc test when appropriate. AKT, protein kinase B; PPARγ, Peroxisome proliferator-activated receptor gamma.

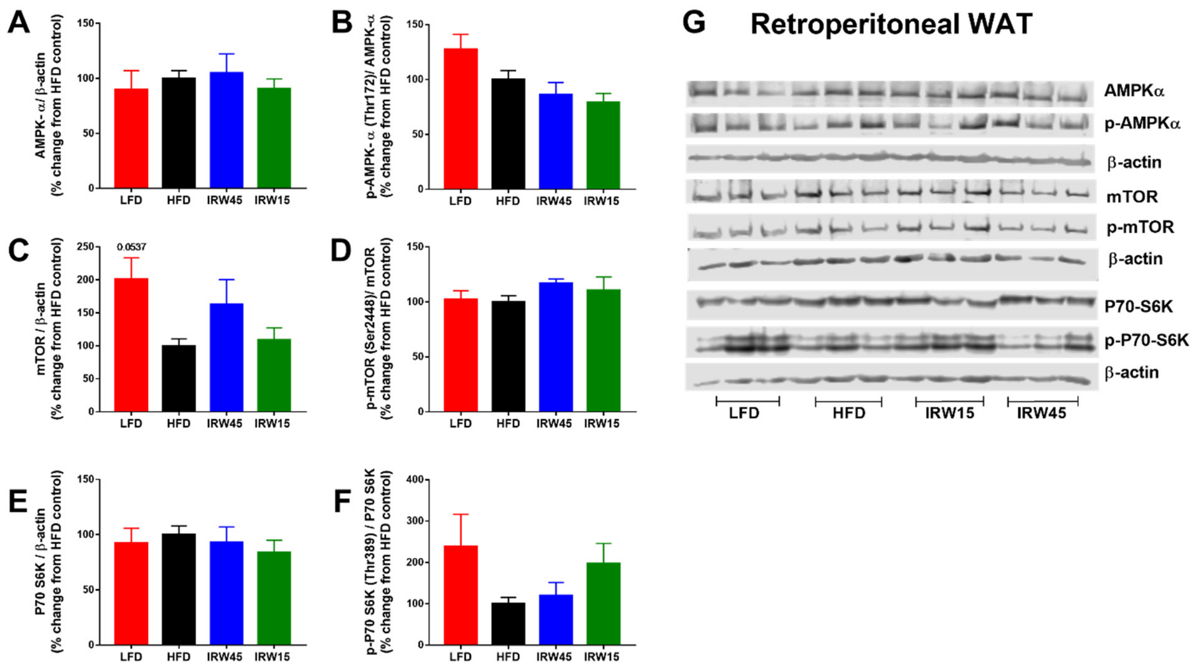

Figure S4: Retroperitoneal white adipose tissue (WAT) AMPKα, mTOR and P70 S6K protein abundance. (A)AMPKα, **(B)** p-AMPKα **(C)** mTOR, **(D)** p-mTOR, **(E)** P70 S6K, **(F)** p-P70 S6K and **(G)** representative blots. Phospho proteins were normalized by their respective total protein. Total protein was normalized to β-actin as the loading control. Data expressed as mean ± SEM of n=6 mice. Analysis by one-way ANOVA followed by Bonferroni's post-hoc test; or Kruskal-Wallis test followed by Dunn's post hoc test when appropriate. 5' AMP-activated protein kinase; mTOR, mammalian target of rapamycin; P70 S6K, Ribosomal protein S6 kinase beta-1.

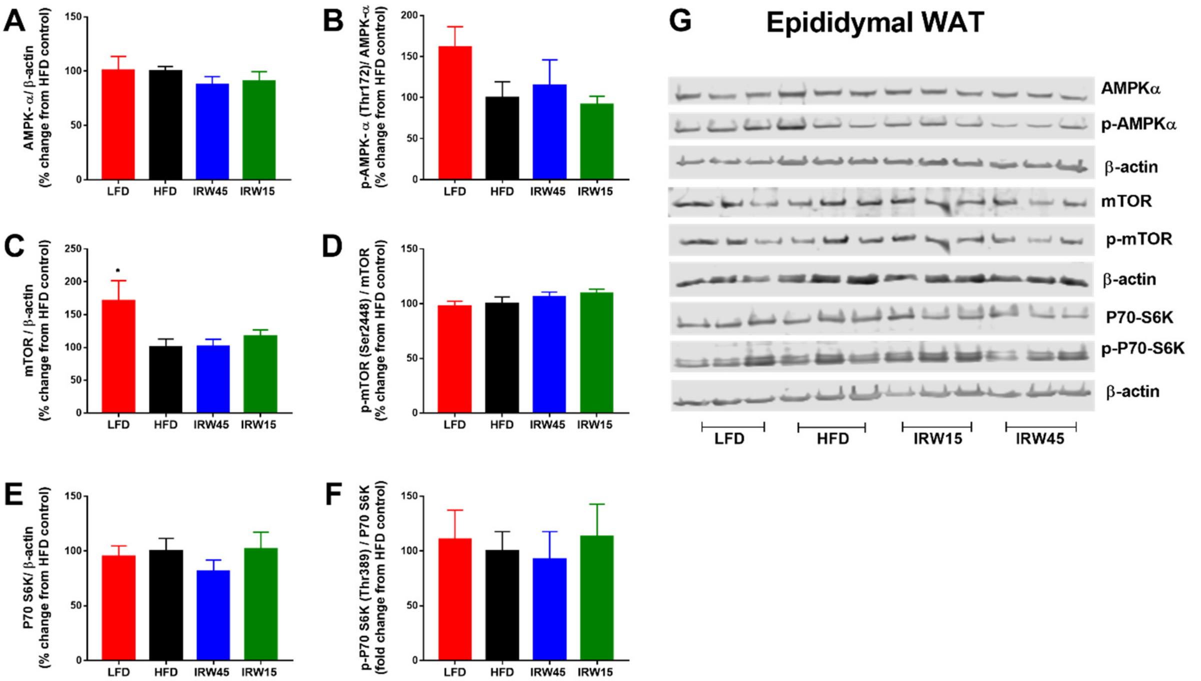

Figure S5: Epididymal white adipose tissue (WAT) AMPKα, mTOR and P70 S6K protein abundance. (A)AMPKα, **(B)** p-AMPKα **(C)** mTOR, **(D)** p-mTOR, **(E)** P70 S6K, **(F)** p-P70 S6K and **(G)** representative blots. Phospho proteins were normalized by their respective total protein. Total protein was normalized to β-actin as the loading control. Data expressed as mean ± SEM of n= 6 mice. Analysis by one-way ANOVA followed by Bonferroni's post-hoc test; or Kruskal-Wallis test followed by Dunn's post hoc test when appropriate.* $p<0.05$ versus HFD. AMPK, 5' AMP-activated protein kinase; mTOR, mammalian target of rapamycin; P70 S6K, Ribosomal protein S6 kinase beta-1. * $p<0.05$ versus HFD.

Table S1. Body composition of C57BL/6 mice after 6 weeks of LFD or HFD feeding.

	LFD (n=7-8)	HFD (n=24)
**BW gain (g)**	8.37±1.0 [a]	9.23±0.4 [a]
**Fat mass %**	22.25 ± 1.5 [a]	29.94 ± 0.7 [b]
**Lean mass %**	70.26 ± 1.5 [a]	63.14 ± 0.7 [b]

Abbreviations: LFD, low fat diet; HFD, high fat diet.

Table S2. Primers sequences used for RT-PCR assays

Gene	Forward Primer	Reverse Primer
**RBM5**	TTGTAATCTGAGTCCCGCCG	CATGGGTGGTATGTTTTAAGGGA
**MDM2**	CCACAGACTAACGACTCGACC	TTTTTGAGGCCCTACTGCGA
**DLG1**	AGATCGCATCATATCGGTGAA	TCAAAACGACTGTACTCTTCGG
**MYOM1**	CAGATGTGTGGCCTCAACTGA	TCGGATTGACTTTGCTCCT
**ASPN**	CTCCAATGTGTGGGTAGGGG	GCACCTGGATCCTAACCCTC
**PPARG**	GGGAAAGACCAGCAACAACC	GCAGTGGAAGAATCGGACCT
**PLIN2**	GAAGGACGTGCAAACAGAAAGG	CTGGGCTATCACGTGGCTCG
**LPL**	GGGAAAGGACTCAGCAGTGTT	TAGGTGGAGGCCACTTCAAGA
**CEBPA**	CCCTTGCTTTTTGCACCTCC	TGCCCCCATTCTCCATGAAC
**GAPDH**	TGAAATGTGCACGCACCAAG	GGGAAGCAGCATTCAGGTCT
